# Biochemical and immunochemical characterization of venoms from snakes of the genus *Agkistrodon*

**DOI:** 10.1016/j.toxcx.2019.100013

**Published:** 2019-08-02

**Authors:** Luis Román-Domínguez, Edgar Neri-Castro, Hilda Vázquez López, Belem García-Osorio, Irving G. Archundia, Javier A. Ortiz-Medina, Vera L. Petricevich, Alejandro Alagón, Melisa Bénard-Valle

**Affiliations:** a Instituto de Biotecnología, Universidad Nacional Autónoma de México. Av. Universidad # 2001 Colonia Chamilpa. CP: 62210. Cuernavaca, Morelos, Mexico; b Facultad de Ciencias Biológicas, Universidad Autónoma del Estado de Morelos. Av. Universidad # 2001 Colonia Chamilpa. CP: 62210. Cuernavaca, Morelos, Mexico; c Campus de Ciencias Biológicas y Agropecuarias, Universidad Autónoma de Yucatán. Km 15.5, Carretera Mérida-Xmatkuil. C.P: 97315. Mérida, Yucatán, Mexico; d Unidad de Manejo para la Conservación de la Vida Silvestre Tsáab Kaan. Km. 2.8, Carretera Baca-Dzemul, C.P. 97450. Baca, Yucatán, Mexico; e Facultad de Medicina. Universidad Autónoma del Estado de Morelos. Calle Leñeros S/N, Colonia Vista Hermosa. CP: 62290. Cuernavaca, Morelos, Mexico

**Keywords:** *Agkistrodon* venoms, Antivenom neutralization, Edema, Hemorrhage, Immunochemistry

## Abstract

In the present work, venoms from five species of the genus *Agkistrodon* were evaluated in terms of their enzymatic (Phospholipase A_2_ and caseinolytic) and biological (edema forming, hemorrhagic, procoagulant and lethal) effects. Horses were used to produce monovalent hyperimmune sera against each of three venoms (*A. bilineatus, A. contortrix* and *A. piscivorus*) and their neutralizing potency, expressed as Median Effective Dose (ED_50_), was determined against the venoms of all five species. In terms of PLA_2_ and caseinolytic activities, all venoms are extremely homogeneous. PLA_2_ activity is high, while caseinolytic activity is low when in contrast with that of the rattlesnake *Crotalus simus*. On the other hand, biological activities showed marked interspecific differences, particularly between the species from Mexico and those from the United States. Mexican species displayed higher edema-forming, hemorrhagic and lethal effects than US species, while none of the species studied presented procoagulant activity. All three monovalent hyperimmune sera showed good neutralizing potency against the analyzed venoms. Nonetheless, we observed relevant immunochemical differences among the venoms using ELISA and Western Blot assays. We conclude that the venoms of *A. piscivorus* (USA) and *A. bilineatus* would be ideal to use as immunogens for the production of a polyvalent antivenom with good neutralizing potency against the venoms of all the species of the genus.

## Highlights

• Venoms from *Agkistrodon* species from the US and Mexico are significantly different in terms of biological activities.
• Edema, hemorrhage and procoagulant, as well as enzymatic activities are determined for five species of the genus.
• Neutralization by three monospecific hyperimmune sera show there is significant cross reactivity between species.
• Venoms from *A. bilineatus* and *A. piscivorus* are proposed as immunogens to generate a genus-wide neutralizing serum.

## Graphical abstract

**Figure:**
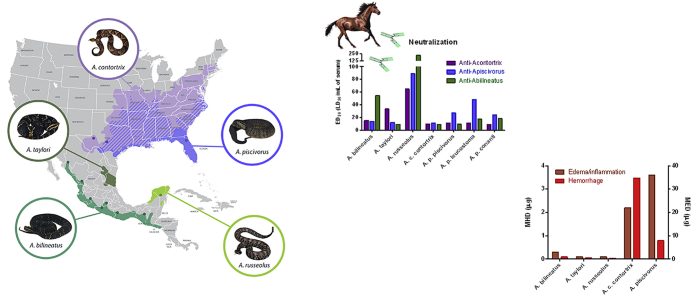

## 1 Introduction

**Fig. 1:**
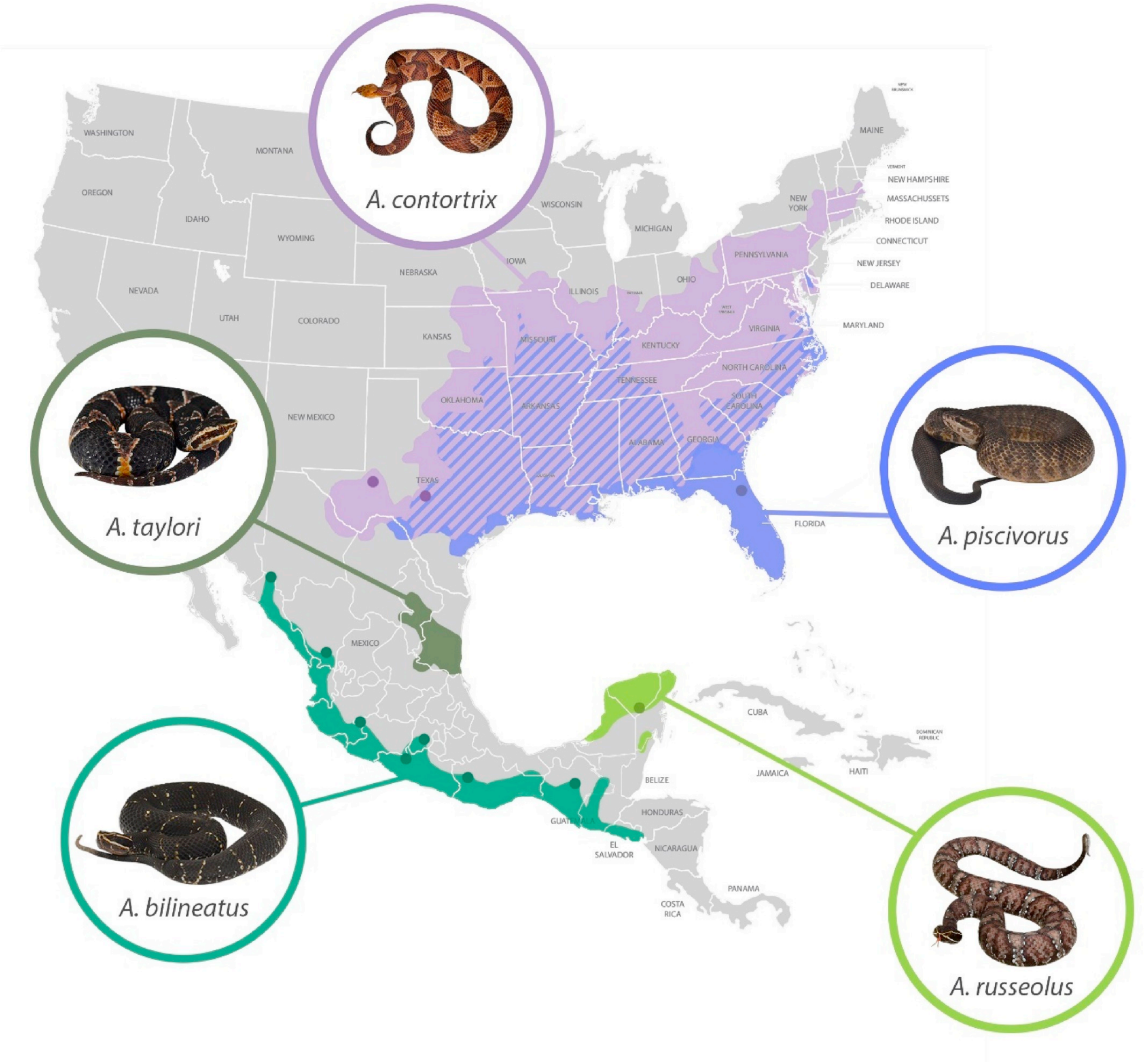
**Distribution of species of the genus *Agkistrodon* in North America.** Colored areas represent the distribution of the species modified from (Campbell and Lamar, 2004, Porras et al., 2013). Diagonal lines represent areas where both *A. contortrix* and *A. piscivorus* are present. *A. c. contortrix* photo by Eric Centenero. (This map is not to scale; it is only meant for illustrative purposes).

Subfamily Crotalinae is a group of snakes, within the family Viperidae, containing about 242 species grouped in 21 genera (Uetz et al., 2019). Snakes of this subfamily, also known as pit vipers, include some Asian genera as well as all the American Vipers; among the latter is the genus *Agkistrodon*. Campbell and Lamar, in 2004, reported 4 species in the genus: *A. bilineatus* (3 subspecies), *A. contortrix* (5 subspecies), *A. piscivorus* (3 subspecies) and *A. taylori*. Later, in 2013, Porras and collaborators elevated the subspecies of *Agkistrodon bilineatus* to species level: *A. bilineatus, A. russeolus* and *A. howardgloydi*, leaving the genus with six species distributed in North and Central America (Fig. 1) (Campbell and Lamar, 2004, Porras et al., 2013). The equine hyperimmunization protocols described in the present study were performed in 2014, and therefore this classification is used throughout the work. Nonetheless, it is important to note that in 2015, some of the subspecies within *A. piscivorus* and *A. contortrix* were elevated to species, leaving four species for the North American *Agkistrodon*: *A. contortrix*, *A. laticinctus, A. piscivorus* and *A. conanti.* For details on the new taxonomy see (Burbrink and Guiher, 2015).

In the United States, there are approximately 45,000 snakebites in humans every year. Among these, about 8,000 result in envenomations. Almost 2,000 are caused by snakes of the genus *Agkistrodon* (Dart and Gomez, 1996)*,* making it one of the most medically relevant in the country. Accurate records regarding snakebite accidents in Mexico are very scarce, but it has been reported that about 4,000 envenomations occur per year, with the genus *Agkistrodon* also being one of the most medically significant (Chippaux, 2017).

The clinical syndrome caused in the USA by the copperhead (*A. contortrix*) is characterized by local symptoms, mainly pain, edema and ecchymosis. Permanent loss of function, necrosis and systemic symptoms are unusual (Scharman and Noffsinger, 2001). These envenomations are thus considered of low risk and antivenom is not always indicated (Mazer-Amirshahi et al., 2014, Walker and Morrison, 2011). Other species of the genus have been reported to cause much more severe envenomations, however. Venom of the Mexican cantil (*A. bilineatus*), for example, in addition to local edema and pain, can cause severe hemorrhages in experimental envenomations (Ownby et al., 1990).

In experimental envenomations, the main activities described for *A. bilineatus* are the generation of hemorrhage and edema and various hemorrhagic toxins have been isolated from *Agkistrodon* venoms (Imai et al., 1989, Ownby et al., 1990). The edema-forming activity of the venoms has been attributed to protein families including phospholipases A_2_ (PLA_2_s), snake venom metalloproteases (SVMPs) and snake venom serine proteases (SVSPs) (de Freitas Oliveira et al., 2009, Hati et al., 1999, Serrano, 2013).

Like most viper venoms, *Agkistrodon* venoms are composed mainly of proteins and peptides while non-proteic components are in lower proportion and include citrate, as well as various ions. Lomonte and collaborators (Lomonte et al., 2014)*,* performed a proteomic analysis of the venoms from four species of the genus *Agkistrodon* and some of their subspecies: *A. contortrix* (five subspecies), *A. piscivorus* (three subspecies), *A. bilineatus* (two subspecies) and *A. taylori*. In that work, they reported that all the venoms have a high proportion of PLA_2_s (31.5–46.0%) and SVMPs (21.0–33.1%), followed by a lower but still important percentage of SVSPs (8.9–22.5%). Together, these three families account for over 60% of the venoms’ components, the rest is composed of other enzymes like L-amino acid oxidases (LAAOs) and non-enzymatic proteins like desintegrins, Cysteine-rich secretory proteins (CRISP) and C-type lectins (CTLs). The proportion of protein families present in the venoms was observed to be very similar, which suggests that they are very homogeneous in terms of composition. Still, the great diversity present within each protein family, complicates the prediction of biological activities or clinical syndromes, even when the proportion of protein families in a venom is known (Castro et al., 2013). Also, the abovementioned differences in severity of the clinical syndromes developed by species of this genus suggest differences in toxicity of individual proteins that are relevant during envenomation and possibly also for antivenom neutralization.

The aim of this work was to characterize the biochemical and biological activities of the venoms of five species of the genus *Agkistrodon*, as well as their immunochemical characteristics. This knowledge can be of importance in the development of antivenoms and in clinical management of envenomation.

## 2 Materials and methods

### 2.1 Ethics statement

All animal experiments were performed in compliance with the EU Directive 2010/63/EU for animal experiments (European Parliament, 2010), under the procedures and with the approval of the Institutional Bioethics Committee of the Biotechnology Institute of the National Autonomous University of Mexico (IBt-UNAM) project number 254: “Functional characterization of *Agkistrodon* venoms and equine immune response against them” (*Caracterización funcional del veneno de Agkistrodon, así como la respuesta inmune en caballos contra los mismos*).

ICR mice used in all experiments were obtained from the laboratory animal facility of IBt-UNAM. Animal facility staff as well as research staff was trained in the correct and humane handling of mice before the start of any procedure.

The use of human blood from a single healthy donor was also approved by the Institutional Bioethics Committee of IBt-UNAM, as a part of project 254. All residues that had been in contact with the blood were discarded in accordance to Institutional regulations.

Some individuals from the species *A. bilineatus* were collected under license number SGPA/DGVS/03459/15 and kept for successive venom extractions at the vivarium “*Cantil: Herpetario del IBt*” with registration number MOR-IN-166-07-04. All housing and handling procedures have been revised and approved by Secretaría de Medio Ambiente y Recursos Naturales (SEMARNAT), Mexico in strict accordance with the regulations of the National General Law of Wildlife (Congreso, 2018).

Horse housing and handling protocols were in strict accordance with Mexican and international animal welfare regulations and were approved by SAGARPA (Secretaría de Desarrollo Agrícola y Desarrollo Rural, Pesca y Alimentación; SENASICA –Dirección General de Salud Animal-) with permit number: DU00411. All handling of horses was performed by previously trained staff from ranch *Ojo de Agua* in Puebla, Mexico and submitted to monthly medical check by an in-house specialized veterinarian.

### 2.2 Venoms

Some venoms were from the venom bank of our laboratory at IBt-UNAM while others were obtained through collaborations with the following herpetariums, who kindly lent their snakes for venom extraction: Reptiles Fergo (license number: DGVS-PIMVS-EA-0084-MOR/08), UMA TSÁAB KAAN (license number UMA-IN-0183-YUC-10), Herpetario de la Facultad de Ciencias, UNAM and DeVal Animal (license number DGVS-CR-IN-0957-D.F./07). Pools from 2 to 5 individuals were used for each species, except in the case of *A. taylori*, where only one specimen was available for venom extraction. Table 1 and Fig. 1 detail the source of all the venoms analyzed in the present study. A pool from 18 adult specimens of *Crotalus simus* from Veracruz, Mexico, and a venom pool from the scorpion *Centruroides limpidus* (both from the venom bank at IBt-UNAM) were used for comparison or external controls when needed.

**Table 1:** Individual venoms used for characterization and hyperimmunization pools.

A. Venom characterization Pools				
Pool	Species	Herpetarium	State of origin	Venom dry weight (mg)
*A. bilineatus*	*A. bilineatus*	DeVal Animal	Colima, MX	8
	*A. bilineatus*	DeVal Animal	Nayarit, MX	8
	*A. bilineatus*	IBt-UNAM	Nayarit, MX	8
	*A. bilineatus*	FC-UNAM	Chiapas, MX	8
	*A. bilineatus*	FC-UNAM	Chiapas, MX	8
*A. taylori*	*A. taylori*	IBt-UNAM	Tamaulipas, MX	6
*A. russeolus*	*A. russeolus*	IBt-UNAM	Yucatán, MX	3
	*A. russeolus*	IBt-UNAM	Yucatán, MX	3
*A. piscivorus*	*A. p. conanti*^a^	NNTRC	Texas, US	1.5
	*A. p. conanti*	“TSÁAB KAAN”	Florida, US	1.5
	*A. p. conanti*	“TSÁAB KAAN”	Florida, US	1.5
	*A. p. leucostoma*^a^	NNTRC	Texas, US	1.5
	*A. p. piscivorus*	“Reptiles Fergo"	Unknown	1.5
*A. c. contortrix*	*A. c. contortrix*^a^	NNTRC	Texas, US	6

B. Horse hyperimmunization Pools				
Pool	Species	Institution	Collection site	Venom dry weight (mg)
Imm-Abil	*A. bilineatus*	IBt-UNAM	Colima, MX	8
	*A. bilineatus*	IBt-UNAM	Sinaloa, MX	8
	*A. bilineatus*	IBt-UNAM	Nayarit, MX	8
	*A. bilineatus*	FC-UNAM	Chiapas, MX	8
	*A. bilineatus*	FC-UNAM	Chiapas, MX	8
Imm-Acont	*A. c. contortrix*^a^	NNTRC	Texas, US	36
Imm-Apisc	*A. p. piscivorus*	“Reptiles Fergo"	Unknown	12
	*A. p. conanti*^a^	NNTRC	Texas, US	12
	*A. p. leucostoma*^a^	NNTRC	Texas, US	12

a Venom pool.

All venoms were obtained through manual extraction and were then washed from the extraction cup using low volumes of 20 mM ammonium acetate pH 4.7 (maximum proportion of ammonium acetate to venom was 1:10 v/v). They were subsequently centrifuged at 16,800 g for 3 min and the supernatant was stored at −70 °C for lyophilization. Finally, lyophilized venoms were stored at 4 °C until their use. Some of the venoms from *A. p. conanti* and all *A. c. contortrix* and *A. p. leucostoma* were pools purchased from the National Natural Toxin Research Center (NNTRC) in Texas, U.S.

### 2.3 Protein concentration

Protein concentration of the pooled and individual venoms was determined using a Pierce® Bicinchoninic Acid (BCA) Protein Assay (Thermo Scientific), with bovine serum albumin (BSA) as a standard, according to the manufacturer's protocols.

### 2.4 Biochemical characterization

#### 2.4.1 SDS-PAGE

Twenty-five μg of each venom were loaded on 12.5% SDS-PAGE gels under reducing conditions. Samples were diluted using Sample buffer 5X (10% Glycerol, 2.5% SDS, Tris-HCl 50 mM pH 6.8, 5% 2-mercaptoethanol, 0.002% bromophenol blue) to a final volume of 20 μL and boiled for 5 min. Electrophoresis was performed with a constant voltage of 80 V for 15 min and then 100 V for approximately 60 min. Gels were stained with G-250 Coomassie Brilliant Blue. Apparent molecular weights were determined comparing migration distance with 5 μL of molecular weight markers (Precision Plus Protein Dual Xtra Standards, Bio-Rad) using ImageJ software version 1.50i.

#### 2.4.2 RP-HPLC profiles

Venom samples (1 mg of dry weight) were dissolved in 1 mL of water containing 0.1% trifluoroacetic acid (TFA), centrifuged to remove debris and fractionated through RP-HPLC on a C_18_ column (4.6 × 250 mm, 5 μm particle size; Vydac®) using an Agilent 1100 chromatograph. Elution was performed at 1 mL/min by applying a gradient to solution B (acetonitrile, containing 0.1% TFA), as follows: 0% B for 5 min, 0–15% B over 15 min, 15–45% B over 60 min, 45–70% B over 12 min, and 70% B for 10 min.

#### 2.4.3 PLA_2_ activity

PLA_2_ enzymatic activity of pooled venoms was determined using a titrimetric assay with a 10% egg yolk solution (0.1 M NaCl, 0.01 M CaCl_2_, 0.1% Triton-100 and 10% egg yolk) as substrate (Shiloah et al., 1973). The assay was performed on 500 μL of the previously described solution, stabilized in pH 8.05 with 50 mM NaOH. The solution was under constant stirring and mild N_2_ bubbling. 50 mM NaOH was also used for titration. Units of enzymatic activity (U) were defined as μmoles of NaOH consumed per minute and the results were reported in units per milligram of venom (U/mg).

#### 2.4.4 Proteolytic activity

Proteolytic activity of the venoms was evaluated using a further modification of the method described by (Chen et al., 2004, Yang et al., 2015) and modified by (Gutiérrez et al., 2008). Briefly, azocasein was dissolved in a standard solution (50 mM Tris-HCl, 150 mM NaCl and 5 mM CaCl_2_) to a final concentration of 10 mg/mL. Afterwards, 20 μg of venom, dissolved in 20 μL of 150 mM NaCl, were added to 100 μL of the azocasein solution and incubated for 30 min at 37 °C. After incubation, the reaction was stopped by adding 200 μL of 5% trichloroacetic acid. Then, samples were centrifuged at 16,800 g for 5 min and 150 μL of the supernatant of each sample were added to 150 μL of 500 mM NaOH in a 96 well plate (NUNC). Finally, sample absorbance at 450 nm was determined. Units of enzymatic activity (U) are defined as the change of 0.2 in the absorbance of the sample per minute.

In order to verify the linearity of the observed reaction, proteolytic activity was determined through varying incubation times (30, 60 and 90 min). The selected incubation time was 30 min, because substrate was still in excess.

### 2.5 Biological characterization

#### 2.5.1 Lethality

The median lethal dose (LD_50_) of each venom pool was determined intravenously (i.v.). Different venom doses in a total volume of 0.5 mL were inoculated through the tail vein to groups of five ICR mice between 18 and 20 g of body weight (4 groups per venom were used in average). The percentage of dead mice was measured 24 h after venom injection with monitoring intervals of approximately 3 h; mice that were evidently moribund were euthanized through cervical dislocation to minimize animal suffering. The obtained data were analyzed through a non-linear regression (variable slope, dose-response curve) using the software GraphPad Prism 6.01. LD_50_ was defined as the amount of venom that causes death to 50% of the mice population (Casasola et al., 2009).

#### 2.5.2 Edema-forming activity

The edema-forming activity of the venom pools was analyzed through the determination of a Minimum Edema-forming Dose (MED), using the method described by (Gutiérrez et al., 1986). Throughout these experiments, groups of three ICR mice were subcutaneously (*s.c.*) injected in the left hind paw with different amounts of venom (4 groups per venom were used in average). Concentrations were calculated in order to always inoculate them with a volume of 50 μL of venom, resuspended in PBS. The right hind paw of every mouse was injected with 50 μL of PBS to use as individual control.

After venom inoculation, the diameter of both paws was measured every 10 min for the first hour and every 30 min for the next 2 h using a manual Vernier caliper. The increase in limb volume caused by the venom for each measured time was determined using the percentage of diameter increase of the envenomated paw compared to the control paw. In order to minimize animal suffering, all mice were euthanized immediately after conclusion of the experiment.

#### 2.5.3 Procoagulant activity

The procoagulant activity of the venom pools was analyzed through the determination of a Minimum Procoagulant Dose in Plasma (MPD-P), using the method described by (Theakston and Reid, 1983). Different amounts of venom were added to glass tubes with 200 μL of citrated (sodium citrate, 3.8 g/dL) human plasma. Time was measured between venom addition and evident clot formation while gently moving the glass tube. Obtained data was processed with a linear regression, selecting only the initial section of the dose-response curve and verifying linearity (R^2^ > 0.9). The venom dose that generates a clot in 1 min was interpolated using the software GraphPad Prism 6.01. The MPD-P was defined as the amount of venom that induces the generation of an evident clot in 60 s.

In order to minimize variation, blood from the same human donor was used for all the experiments and the time measurements were always taken by the same observer.

#### 2.5.4 Hemorrhagic activity

The hemorrhagic activity of the venom pools was analyzed through the determination of a Minimum Hemorrhagic Dose (MHD) using the method by (Gutiérrez et al., 1985) with some modifications. Briefly, groups of 5 CD1 mice were intradermally (*i.d.*) inoculated in the higher region of the back with 50 μL of venom resuspended in PBS with varying concentrations (4 groups per venom were used in average). Three hours after inoculation, mice were sacrificed through CO_2_ inhalation and their skins were removed. The hemorrhagic area (HA) around the injection point was measured using millimetric paper and the diameter (D) of the hemorrhagic halo was calculated using the following formula: $D=\;2\;*\;\sqrt{\frac{HA}{\pi }}$ (Varios, 2007). MHD was defined as the amount of venom that generates a hemorrhagic halo of 1 cm in diameter.

### 2.6 Immunochemical characterization

#### 2.6.1 Production of horse hyperimmune sera

Three adult, male, crossbred horses kept in the farm “Ojo de Agua” in the community Venustiano Carranza (Puebla, Mexico), were inoculated with increasing amounts of venom, using the immunization scheme detailed in Supplementary Table 1. Each horse was identified with a number and inoculated with the venom from only one *Agkistrodon* species as follows: Horse 201 with *A. c. contortrix*, horse 202 with a pool of the three subspecies of *A. piscivorus* and horse 203 with *A. bilineatus*. The hyperimmunization pools used are detailed in Table 1.

#### 2.6.2 Determination of titers of *Agkistrodon* hyperimmune horse sera

Maxisorp (Nunc Inc, USA) plates were coated with 100 μL/well of 5 μg/mL of the venoms*,* diluted in 100 mM carbonate/bicarbonate buffer, with pH 9.5 and incubated overnight at 4 °C. The plates were then washed 3 times with 250 μL/well of washing buffer (50 mM Tris/HCl, 150 mM NaCl, 0.05% Tween 20 and pH 8) in a microplate washer (BIO-RAD Immuno wash 1575). The remaining binding sites were blocked with 200 μL/well of blocking buffer (50 mM Tris/HCL, 5 mg/mL gelatin, 0.2% Tween 20 and pH 8) and incubated for 2 h at 37 °C. The plates were then washed 3 times as described before. Samples of anti-*Agkistrodon* horse serum were initially mixed with vehicle buffer (50 mM Tris–HCl, 0.5 M NaCl, 1 mg/mL gelatin and 0.05% Tween 20, pH 8.0), at a dilution of 1:300. This solution was serially diluted 1:3, with the same buffer on the ELISA plates. Plates were incubated for 1 h at 37 °C and, after washing them 3 times, plates were incubated for 1 h at 37 °C with 100 μL/well of peroxidase-conjugated goat anti horse IgG antibody (1:3000 dilution, Gene Tex). After washing them 5 times, 100 μL/well ABTS solution (Roche) were added and incubated for 10 min at 25 °C. When this timespan concluded, the reaction was stopped with 20 μL/well of 20% sodium dodecyl sulfate (SDS) and absorbances of wells were measured at 405 nm in a Microplate Reader. Sigmoidal dose-response curves were generated using non-linear regression with a variable slope with the software GraphPad Prism 4. Antibody titer is defined as the serum dilution at which 50% of the colorimetric response is obtained.

#### 2.6.3 Competitive ELISA

We used two different plates, incubation and assay plates. Incubation plates (Maxisorp Nunc Inc, USA) were blocked with 200 μL/well of blocking buffer and incubated for 2 h at 37 °C. Samples of *Agkistrodon* venoms were prepared in vehicle buffer with a concentration of 300 μg/mL or 1000 μg/mL and serially diluted 1:3. Also, 100 μL/well of horse serum anti *Agkistrodon* were added to the plate using a dilution equivalent to the titer (EC_50_) of each serum for its homologous venom and incubated for 1 h at 37 °C.

Assay plates were coated with 100 μL/well of 5 μg/mL of venoms from *A. bilineatus, A. c. contortrix* or a mixture of *A. piscivorus* subspecies and incubated overnight at 4 °C. The remaining binding sites were blocked with 200 μL/well of blocking buffer, incubated for 2 h at 37 °C.

The mixture from the incubation plates was then added to the assay plates and these were incubated for 1 h at 37 °C. Finally, plates were incubated for 1 h at 37 °C with 100 μL/well of peroxidase-conjugated goat anti-horse IgG antibody (1.5 10^−3^ μg/mL, Gene Tex) and developed using 100 μL/well of ABTS solution (Roche) incubated for 10 min at 25 °C. After this timespan concluded, the reaction was stopped with 20 μL/well of 20% SDS. Each samples’ absorbance was determined at 405 nm in a Microplate Reader.

The serum titer for the homologous venom was used to define the serum dilution to add as primary antibody to each ELISA plate. The absorbance of the maximum venom dilution of the homologous venom for each plate was defined as 0% competition and that of the highest venom dilution, as the percent of maximum inhibition. Data analysis was performed using the software GraphPad Prism 4.0.

#### 2.6.4 Neutralizing potency

Neutralizing potency of the horse hyperimmune sera was evaluated through the determination of a Median effective Dose (ED_50_) (Casasola et al., 2009). To this end, different serum volumes were incubated for 30 min at 37 °C with 3LD_50_s of *Agkistrodon* venom. Afterwards, groups of 5 mice between 18 and 20 g of body weight were inoculated *i.v.* with the samples (4 groups per venom were used in average). The survival rate was determined 24 h after venom injection with monitoring intervals of approximately 3 h; mice that were evidently moribund were euthanized through cervical dislocation to minimize animal suffering. Obtained data was processed through non-linear regression (variable slope, dose-response curve) using the software GraphPad Prism 6.01. The ED_50_ was defined as the serum volume capable of preventing death in 50% of envenomated mice.

### 2.7 Statistical analysis

In order to evaluate differences between treatments, a one way ANOVA and a *post hoc* Tukey test were performed for PLA_2_ and caseinolytic activities. Results were considered statistically different when P < 0.05.

## 3 Results

### 3.1 Biochemical and biological characterization

**Fig. 2:**
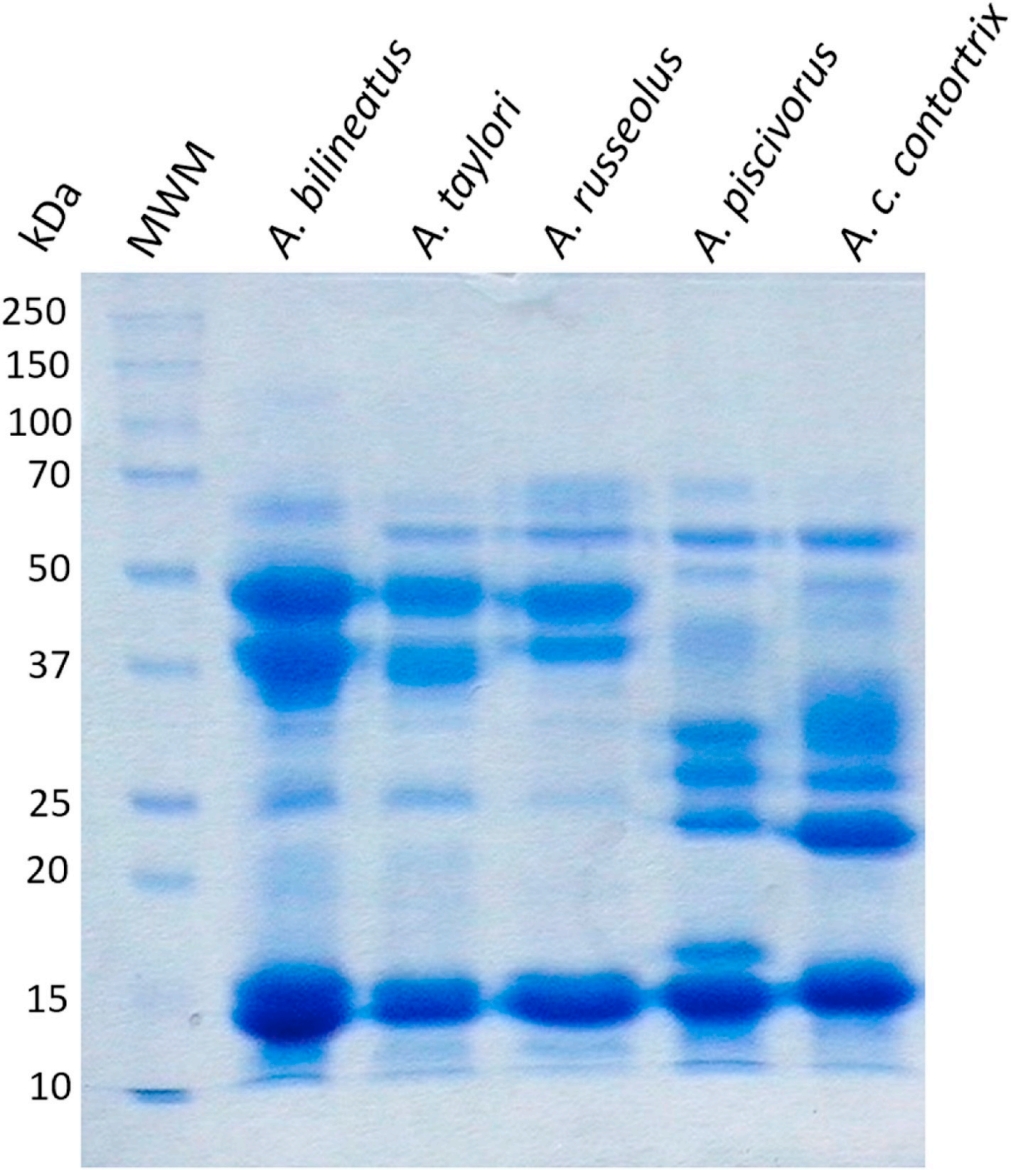
**SDS-PAGE profile of *Agkistrodon* venom pools.** Reducing conditions (2 ME). *MWM.* Molecular weight markers.

Electrophoretic profiles of *Agkistrodon* venoms are shown in Fig. 2. All analyzed venoms showed several conspicuous protein bands of 13.2 kDa–15.3 kDa. Venoms from the Mexican species (lanes 2 to 4) showed very similar patterns among them, with two more abundant protein bands of approximately 35.2 and 45.8 kDa and less abundant bands of around 27.1, 45.8 and 58.3 kDa. On the other hand, electrophoretic pattern of venoms from *A. piscivorus* and *A. c. contortrix* differed from the Mexican venom pattern and were also relatively different from each other. These venoms lacked the abundant high molecular weight proteins, or had them in very small proportion, and were instead rich in medium molecular weight protein bands, ranging from 24.9 to 35 kDa (three bands in *A. piscivorus* and four in *A. c. contortrix*).

**Fig. 3:**
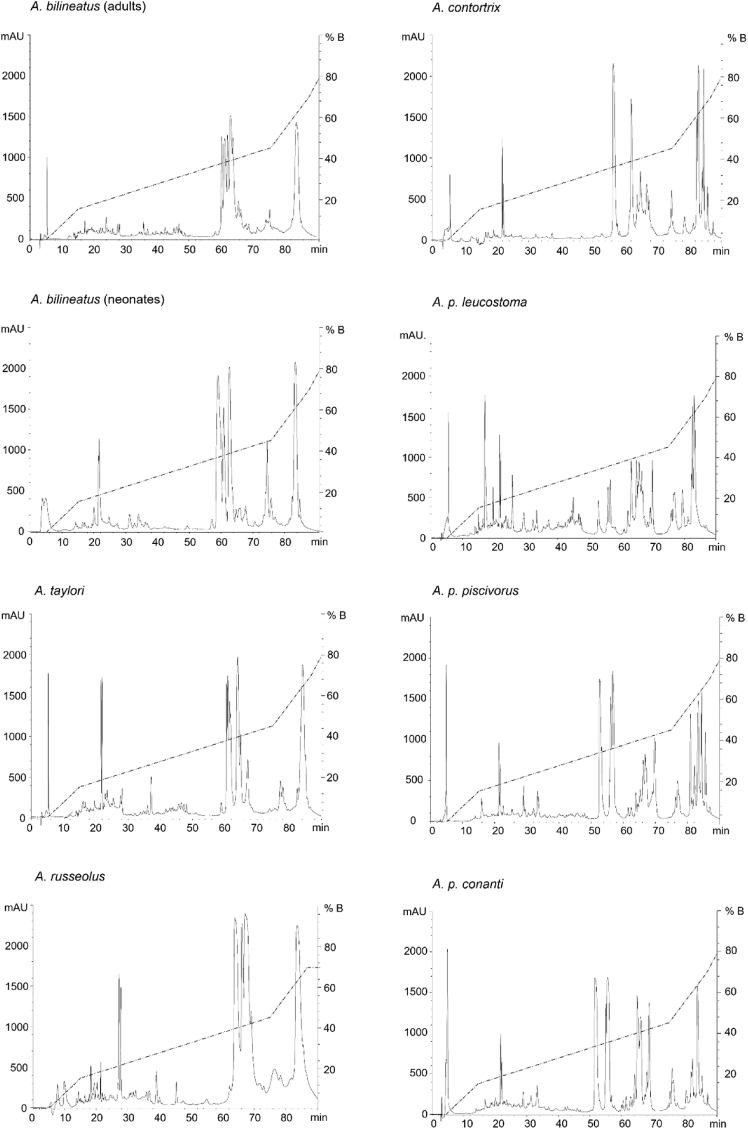
**RP-HPLC of *Agkistrodon* venom pools on a C**_**18**_**analytic column.***mAU.* Milli Absorbance Units at 214 nm *% B.* Percentage of buffer B (CH_3_CN + 0.1% TFA).

RP-HPLC profiles also showed the Mexican species to be very similar to each other, with the most abundant components eluting between 60 and 70 min of retention time (RT) and a single, also very abundant peak at approximately 85 min. Profiles from *A. c. contortrix* and the subspecies of *A. piscivorus* were similar, but not identical, with some marked differences before 50 min RT; both species differed significantly from the profiles of the Mexican species. Some of the observed differences were a greater diversity of components around 85 min RT and the presence of one to three abundant components between 50 and 60 min RT (Fig. 3).

In Table 2 we summarized the results obtained for the biochemical and biological activities of *Agkistrodon* venoms. The venom of *C. simus* was used for comparison where relevant.

**Table 2:** Enzymatic and pharmacological activities of *Agkistrodon* venom pools.

	PLA_2_^a^	Proteolysis^b^	MED^c^	MHD^d^	MPD-HP^e^	LD_50_^f^	
	(U/mg ± SD)^g^	(U/mg ± SD)^g^	(μg ± SD)	(μg ± SD)	(μg)	(μg/mouse)	(μg/g)
*A. bilineatus*	383.2 ± 97.7	6.8 ± 1.3	0.2 ± 0.01	0.8 ± 0.04	>100	36.5 (36–37.1)	1.9
*A. taylori*	720.2 ± 81	6.4 ± 0.6	0.1 ± 0.01	0.7 ± 0.25	>100	26.2 (22.7–30.6)	1.4
*A. russeolus*	371.5 ± 146.3	5.2 ± 0.7	0.1 ± 0.03	0.3 ± 0.06	>100	22.3 (22.2–22.3)	1.2
*A. c. contortrix*	296.8 ± 64.9	7.0 ± 1.0	1.8 ± 1.2	33.8 ± 1.4	>100	215.4 (197.0–235.5)	11.3
*A. piscivorus*	231.9 ± 49.3	6.4 ± 0.6	3.6 ± 0.5	7.4 ± 0.74	>100	96.6 (92.6–100.9)	5.1
*C. simus*	ND^h^	11.2 ± 1.6	10.0 ± 1.2	25.1 ± 1.3^i^	ND^h^	3.8 (3.4–3.9)	0.2

a Phospholipase A_2_ activity on 10% egg yolk.

b Proteolytic activity on azocasein.

c Minimum edema-forming dose.

d Minimum hemorrhagic dose.

e Minimum procoagulant dose on human plasma.

f Median lethal dose, values in parenthesis represent 95% confidence intervals.

g Units of enzymatic activity per milligram of venom ± standard deviation.

h Not determined.

i (Castro et al., 2013).

LD_50_s of the analyzed venoms ranged from 0.2 to 11.3 μg/g and are shown in Table 2. In terms of PLA_2_ activity, most *Agkistrodon* venoms showed no statistically significant difference between them (P ≥ 0.05), ranging between 231.9 and 383.2 U/mg; *A. taylori* was the only one with a significantly higher activity (720.2 U/mg). Proteolytic activity on azocasein substrate was also statistically the same for all the *Agkistrodon* venoms tested (P ≥ 0.05), ranging between 5.2 and 7 U/mg (Table 2).

In the current work, we observed that edema-forming activity was greatest in the venoms from *A. bilineatus, A. taylori* and *A. russeolus,* with MED ranging between 0.1 and 0.2 μg. In contrast, the venoms from *A. c. contortrix* and *A. piscivorus* showed MEDs of 1.8 and 3.6 μg respectively; between 7.3 and 36.0 times higher than those of the Mexican species (Table 2). Also, all venoms tested caused hemorrhage to some extent, yet once again, the venoms from *A. bilineatus, A. taylori* and *A. russeolus* had the highest activities with MHDs of 0.8, 0.7 and 0.3 μg respectively. The venoms from *A. piscivorus* and *A. c. contortrix* had comparatively less activity, with MHDs of 7.4 and 33.8 μg respectively (Table 2).

### 3.2 Immunochemical characterization

#### 3.2.1 ELISA titers

To analyze the recognition of the produced hyperimmune sera, we determined ELISA titers using homologous and heterologous *Agkistrodon* venoms. Highest titers were obtained for serum 203 (anti-*A. bilineatus*) against the two other Mexican species (*A. russeolus* −121,043- and *A. taylori* −122,525-) while titers against *A. c. contortrix* and the subspecies of *A. piscivorus* ranged between 33,522 and 67,872. Interestingly, the titer against the homologous venom was relatively low (37,003) (Table 3).

**Table 3:** Antibody titers and neutralization potency of produced sera against *Agkistrodon* venoms.

	LD_50_^a^	Serum 201 [anti-*A. c. contortrix*]			Serum 202 [anti-*A. piscivorus*]			Serum 203 [anti-*A. bilineatus*]		
		Titer	ED_50_^b^		Titer	ED_50_^b^		Titer	ED_50_^b^	
	μg/g		mgV/mLS^c^	LD_50_/mLS^d^		mgV/mLS^c^	LD_50_/mLS^d^		mgV/mLS^c^	LD_50_/mLS^d^
*A. bilineatus*	1.9	18,467	0.5 (0.5–0.6)	15.8	15,821	0.5 (0.4–0.6)	14.2	**37,003**	**2.0 (1.6–2.4)**	**54.4**
*A. taylori*	1.4	41,131	0.9 (0.9–0.9)	33.5	44,780	0.3 (0.3–0.4)	12.6	122,525	0.2 (0.2–0.3)	9.4
*A. russeolus*	1.2	34,457	1.5 (1.4–1.5)	65.1	49,880	2.0 (1.6–2.4)	89.1	121,043	5.1 (4.9–5.4)	229.5
*A. c. contortrix*	11.3	**29,829**	**2.2 (2–2.3)**	**10.2**	30,528	2.5 (2.1–3.0)	11.8	33,522	2.0 (1.7–2.4)	9.4
*A. p. piscivorus*	5.1	23,278	1.1 (1.0–1.3)	11.8	**110,262**	**2.6 (2.4–2.9)**	**27.3**	43,676	0.9 (0.9–1.0)	9.7
*A. p. leucostoma*	6.1	20,755	1.4 (1.3–1.5)	11.8	**83,123**	**5.7 (4.6–7.0)**	**48.6**	61,035	2.1 (1.6–2.8)	17.7
*A. p. conanti*	6.3	29,829	1.1 (1.0–1.1)	9.0	**108,032**	**2.9 (2.8–3.1)**	**24.5**	67,872	2.2 (2.0–2.4)	18.5
Numbers in parenthesis represent 95% confidence intervals. Values in bold represent responses to the homologous venom.										

a Median lethal dose.

b Neutralization median effective dose, using 3LD_50_s of venom.

c Milligrams of venom neutralized per milliliter of serum.

d Median lethal doses of venom neutralized per milliliter of serum.

Serum 201 (anti–*A. c. contortrix*) had the lowest titers, ranging between 18,467 and 34,457 against the Mexican species and between 23,278 and 29,829 against species from the U.S., including the homologous venom. Finally, titers for serum 202 (anti-*A. piscivorus*) were high against the homologous subspecies (83,123 to 110,262) and relatively low against both *A. c. contortrix* and the Mexican species (15,821 to 49,880) (Table 3). Titers for the negative control venom, *Centruroides limpidus*, were 0 against all sera.

#### 3.2.2 Competitive ELISA

**Fig. 4:**
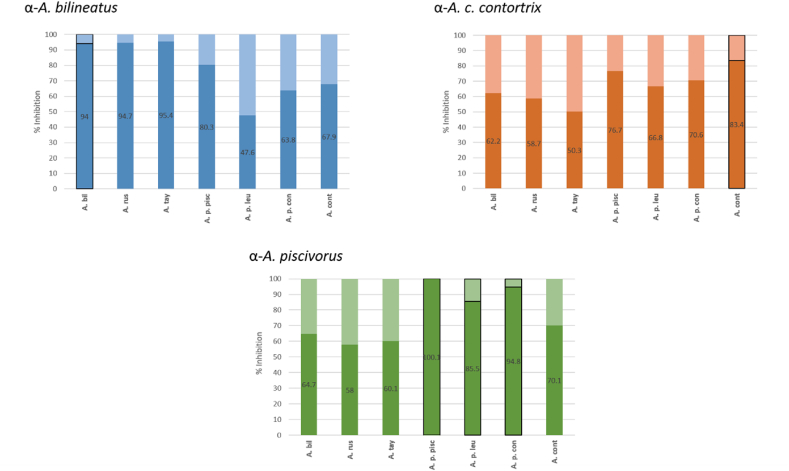
**Percentage of competition of each horse serum with *Agkistrodon* venoms, determined using a competitive ELISA.** A higher inhibition percentage shows more shared epitopes between the homologous and tested venom.

In the plates with *A. bilineatus* venom and serum against *A. bilineatus,* we obtained highest percentages of competition with Mexican species of *A. bilineatus*, *A. russeolus* and *A. taylori,* while less was observed for species from USA, showing that Mexican species are immunochemically similar to each other. On the other hand, on the plate with *A. piscivorus* fixed venom, we obtained the maximum inhibition with homologous *A. piscivorus* venom and a moderate inhibition with *A. c. contortrix* venom (70.1%). The species of *A. bilineatus*, *A. russeolus* and *A. taylori* were bad competitors. Finally, venom from *A. c. contortrix* was the one that showed lowest competition values with all other species. Still, Mexican species share fewer epitopes with it than do the subspecies of *A. piscivorus* (Fig. 4).

There was no competition with the venom of the negative control, *C. limpidus*, in any of the analyzed sera, indicating that there is no non-specific recognition by equine immunoglobulins.

#### 3.2.3 Neutralization

Monovalent sera, in general, presented a higher neutralization potency against the homologous venom than against the heterologous venoms. Serum 201 (anti-*A. c. contortrix*), showed an EC_50_ of 2.2 mgV/mLS (milligrams of venom per milliliter of serum) against the homologous venom, while this same value ranged between 0.6 and 1.5 mgV/mLS when tested against heterologous venoms. Serum 202 (anti-*A. piscivorus*) presented a neutralization potency between 2.6 and 5.7 mgV/mLS for the three subspecies of *A. piscivorus* and between 0.3 and 2.5 mgV/mLS for other species of the genus. On the other hand, serum 203 (anti-*A. bilineatus*) was an exception because it neutralized the venoms from *A. russeolus, A. c. contortrix, A. p. leucostoma* and *A. p. conanti* just as well or better than the homologous venom (5.1, 2.0, 2.1 and 2.0 mgV/mLS, respectively) (Table 3).

## 4 Discussion

General composition in viper venoms is broadly conserved in terms of protein families (Calvete et al., 2009, Lomonte et al., 2014), where the most abundant are usually SVMPs, PLA_2_s and SVSPs (Tasoulis and Isbister, 2017). Differences among the species of a genus such as *Agkistrodon* are generally given by variation in the proportions of the mentioned protein families and by the presence or absence of individual proteins which can have a strong effect in a particular venom activity (Borja et al., 2018, Castro et al., 2013, Glenn et al., 1994, Saldarriaga et al., 2003). Venom variation can be immunochemical, biochemical or in terms of its toxic activities and it can significantly affect neutralization by antivenoms (de Roodt et al., 2011, Gutiérrez et al., 2010, Neri-Castro et al., 2019). It has been shown that it can exist between species and within a single species, generated by ontogenetic changes or from one geographic location to another, among other factors (Borja et al., 2018, Borja et al., 2014, Borja et al., 2013, Castro et al., 2013, Durban et al., 2017, Glenn et al., 1994, Glenn et al., 1982, Mackessy et al., 2018, Rivas et al., 2017, Wilkinson et al., 2018, Zancolli et al., 2018, Zelanis et al., 2010).

Only interspecific variation was considered in the present study, while the possibility of the existence of intraspecific variation in *Agkistrodon* venoms was not evaluated, mainly due to sample availability. However, samples from a wide distribution range were available for the case of *A. bilineatus* (Fig. 1), so biochemical activities as well as SDS-PAGE were performed with 12 individual venoms. We observed that there is no evident intraspecific variation across the distribution range of this species (Supplementary Figure 1). Also, a pool of 9 neonate individuals from *A. bilineatus* (born in captivity) was analyzed using the same RP-HPLC method used for the pooled venoms and it proved to be almost identical to the adult pool (Fig. 3). Intraspecific variation has been also analyzed using the venom of *A. contortrix* by (Lagesse and Ford, 1996) and found the venom of this species to have only small variations across its distribution.

In the SDS-PAGEs performed in the present work, we observed that the Mexican species have similar electrophoretic profiles among them and are different from the species native to the U.S. (Fig. 2). This grouping can be also observed in the RP-HPLC profiles (Fig. 3). Except for a few small differences, the RP-HPLC profiles were extremely similar to the ones previously obtained by Lomonte and collaborators (Lomonte et al., 2014), during their proteomic characterization of these species (Fig. 3).

Proteins of the PLA_2_ families were very abundant in all the venoms studied in the present work. Within the PLA_2_ proteins that have been described in viper venoms there are two distinct groups: those with enzymatic activity, which have an aspartic acid in canonical position 49 (D49), and those with a substitution of this residue to lysine or serine (K49 or S49) and that are devoid of enzymatic activity (Fernández et al., 2013, Kini, 2003, Lomonte et al., 2003). Many among the second group are commonly referred to as true myotoxins because they act on cellular membranes of muscle cells causing lysis through direct damage (Lomonte and Rangel, 2012, Ward et al., 2002). D49 PLA_2_s have extremely variable pharmacological activities, often interfering in cellular signaling cascades or causing cell lysis through breakdown of membrane phospholipids (de Freitas Oliveira et al., 2009). In the particular case of *Agkistrodon* venoms, no relationship was observed between the presence of D49 or K49 PLA_2_s and the edema-forming activity of the complete venoms (Table 4).

**Table 4:** Type of PLA_2_s present in *Agkistrodon* venoms.

	% D49^a^	% K49^a^	MED^b^ (μg)	PLA_2_^c^ (U/mg)
*A. bilineatus*	16.9	17.9	0.2	383.2
*A. taylori*	26.5	7.8	0.1	720.2
*A. c. contortrix*	9.8	21.7	1.8	296.8
*A. p. piscivorus*	24.0	12.8	3.6^d^	
*A. p. leucostoma*	28.6	14.6		231.9^d^
*A. p. conanti*	28.7	17.3		

a Percentage data were obtained from Lomonte et al. (2014).

b Minimum Edema-forming Dose (micrograms).

c PLA_2_ enzymatic activity (Units per milligram).

d Activity determined using a pool of the three subspecies.

All venoms showed a similar proteolytic activity when using azocasein as substrate. This activity is due both by SVMPs and SVSPs (Serrano, 2013). SVMPs are found in similarly high proportions in all the species of the genus *Agkistrodon* as shown in the present work and in (Lomonte et al., 2014). Nonetheless, when performing a deeper analysis of the available proteomes, we observe than the Mexican species have a much higher amount of type P-II SVMPs while the ones present in *A. c. contortrix* and *A. piscivorus* are mainly of the type P–I (Table 5). These differences can also be observed in the electrophoretic profiles (Fig. 2).

**Table 5:** Metalloprotease type in *Agkistrodon* venoms.

Group	SVMPs %	
	*A. c. contortrix*	*A. taylori*
P–I	29.7	1.9
P-II	2.3	28.1
P-III	0.5	0.6

Adapted from Lomonte et al. (2014).

One of the main pharmacological activities that can be caused by SVMPs is hemorrhage (Fox and Serrano, 2005). In 1989, Imai et al. (Imai et al., 1989, Nikai et al., 2000) isolated and described a protein they called Bilitoxin-I (PII-SVMP, MW ≈ 48 kDa) from the venom of *A. bilineatus*. Although P-III SVMP have been described generally to be the most potent hemorrhagic SVMPs (Fox and Serrano, 2005), Bilitoxin-I has an extremely potent hemorrhagic activity, MHD of 0.008 μg, and it is likely responsible for the high hemorrhagic activity observed for the Mexican species (Table 2). For example, *A. taylori*, had a MHD of 0.7 μg and this protein and similar isoforms account for about 28% of total venom (Lomonte et al., 2014). *A. russeolus* has been minimally studied but its low MHD (0.3 μg), an abundant RP-HPLC peak with retention time of 83.6 min (consistent with Bilitoxin-I in *A. bilineatus* and *A. taylori*), and our observation of severe hemorrhages in envenomated mice, indicate Bilitoxin-I could also be present in high proportions in this venom. On the other hand, based on *A. c. contortrix* or *A. piscivorus* venom proteomes (Lomonte et al., 2014), these species contain less than 2% of Bilitoxin-I or similar proteins. These venoms are much less hemorrhagic, with MHDs of 33.8 and 7.4 μg respectively. Hemorrhages observed in these cases may therefore be caused by other, less potent, SVMPs.

Regarding the minimum procoagulant dose in human plasma (MPD-HP), no clot formation was observed for 10 min when adding 100 μg of any venom to 200 μL of citrated human plasma. Procoagulant activity is usually observed in viper venoms that have a high proportion of SVSPs with “thrombin-like” activity as well as other, less common, SVSPs and SVMP that act on different coagulation factors. Examples of these include prothrombin and factor X activators within the SVMP family (Ramos and Selistre-de-Araujo, 2006) and activators of factors V, VIII and also factor X and prothrombin within the SVSP family (Serrano, 2013). One thrombin-like SVSP, named Bilineobin, has been described in the venom of *A. bilineatus* (Komori et al., 1993), though it has low procoagulant activity when compared to that of other viper venoms. In our experiments, the presence of this protein does not appear to be relevant when the whole venom is tested (Table 2), but the formation of some fibrin fibers was observed. Previous reports have also shown a lack of procoagulant activity of various *Agkistrodon* venoms (Arce et al., 2003, Lomonte et al., 2014). Contrasting with this result, procoagulant activity has been previously reported for the venom of *A. bilineatus* (de Roodt et al., 2005), and a Protein C activator protein has been previously described in the venom of the same species (Nakagaki et al., 1990). Some procoagulant enzymes have also been described in the venoms of closely related genus of snakes (Li et al., 2018, Yukelson et al., 1991). On the other hand, a fibrinolytic SVMP without hemorrhagic activity has been described in the venom of *A. contortrix laticinctus* (Selistre De Araujo, 2013)*.*

One of the most clinically conspicuous signs during *Agkistrodon* envenomation is edema and inflammation (Scharman and Noffsinger, 2001). Edema is the extravasation of fluid to the interstitial space, and even though it is often related to an inflammatory process, this is not always the case (Trigo and Valero, 2004). Inflammation is a complex cascade of biochemical and cellular processes initiated and regulated by the immune system. The classic manifestations of an inflammatory event are pain, heat, blushing, and local volume increase. Inflammation can be classified in acute and chronic, primarily based on duration and the presence of healing at the site of inflammation. The first is characterized by being short in duration (generally less than 4 h), usually generated by spontaneous damage, while the second is long in duration and characterized by the initiation of healing (Owen et al., 2014, Trigo and Valero, 2004).

The venom of several snakes has been reported to start a complex inflammatory process, associated with the liberation or synthesis of mediator molecules that recruit different cells, as well as pain and edema (Gutiérrez, 2002). The main protein families that have been described to cause inflammation are SVMPs and PLA_2_s (de Freitas Oliveira et al., 2009, Gutiérrez and Lomonte, 2013, Mackessy, 2009); both groups possess different mechanisms of activation of the immune response. *Bothrops asper* has one of the most studied venoms in this matter. Here, SVMPs favor edema by causing the rupture of blood vessels and therefore the extravasation of liquid to the interstitial space without the previous initiation of an inflammatory process (Rucavado et al., 2002, Rucavado et al., 1995); inflammation will start later in response to tissue damage. On the other hand, some PLA_2_s from this venom generate damage of muscle tissue (Zuliani et al., 2005), promoting an inflammatory response without an initial edema.

**Fig. 5:**
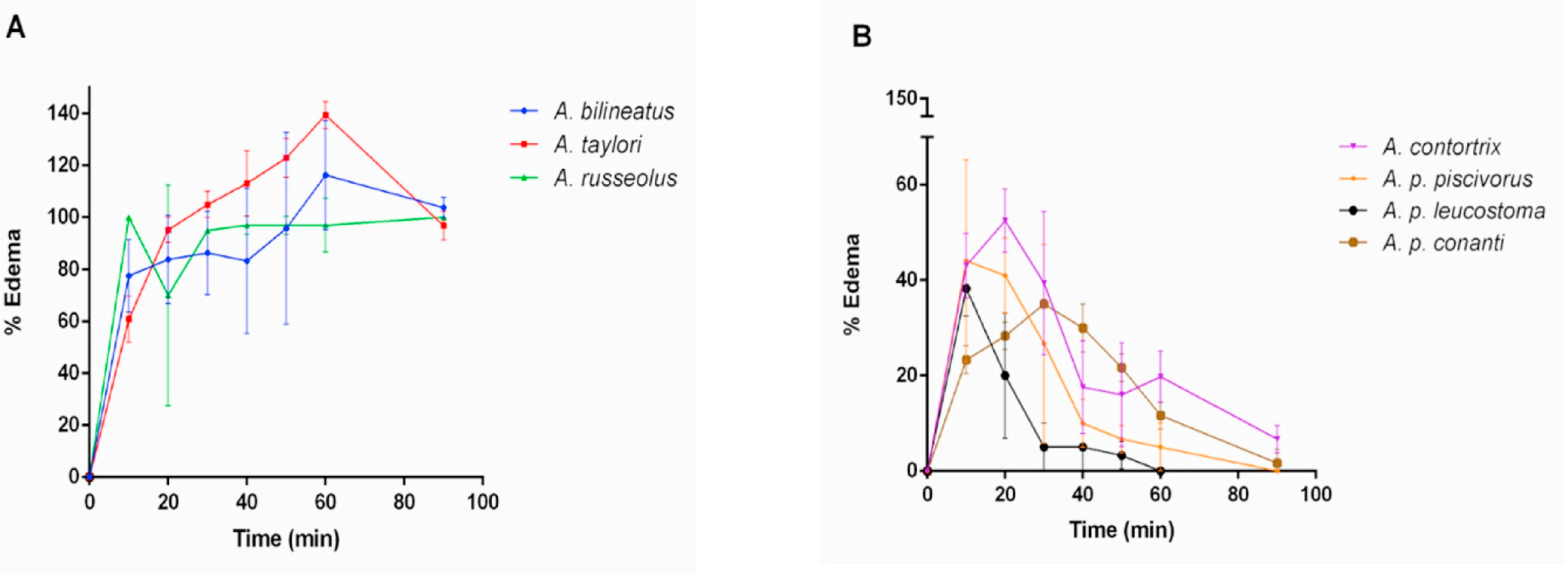
**Progression of edema on mouse paw injected subcutaneously with different *Agkistrodon* venoms from Mexico (A) or the U.S. (B).** Percentage represents increased diameter compared to the contralateral paw (See Materials and Methods).

In the present work, the progression of edema/inflammation was different in both groups of venoms. Injection of venom from the Mexican species resulted in a slow increase in limb volume, with a maximum around 50 min, after which it started decreasing and returned to 0% about 3 h after subcutaneous venom injection (Fig. 5). This behavior could be caused by an initial rupture of the vascular endothelium by SVMPs generating tissue damage and starting an inflammatory response. Also, the venom of *A. bilineatus* has been described to have bradykinin potentiating factors (BPF) which directly increase vascular permeability (Munawar et al., 2016).

On the other hand, both species from the U.S. showed a rapid increase in limb diameter, with a maximum at 10 min and a relatively fast decrease, returning to 0% around 2 h after inoculation (Fig. 5). This suggests the extravasation of liquid without the initiation of an intense inflammatory process.

More detailed studies, including histology of envenomated tissue and/or quantification of inflammation mediators, should be done in order to obtain a proper description of this process.

Another important factor to discuss is the individual variation in humoral immune response of horses used for hyperimmunization. It has been previously observed that horses submitted to the same immunization protocol and adjuvants can have different responses in terms of antibody titers as well as neutralization potencies against homologous venoms (Angulo et al., 1997, Calderón Corona, 2011, Estrada et al., 1989). In this work, only one horse per venom was used for the production of hyperimmune sera and therefore we have no way of verifying that the differences observed are due to the venom's properties as antigens and not to individual variation between horses. Further studies must be done in order to address this issue.

The venoms from Mexican species of this genus were more lethal to mice than those of both species from the U.S. (Table 2). Similar lethal potencies, of 4.6 and 9.1 μg/g, have been previously determined for *A. piscivorus* and *A. c. contortrix*, respectively (Arce et al., 2003). The high lethality of the Mexican species could also be explained by the presence of the aforementioned Bilitoxin-I, whose reported LD_50_ is 0.5 μg/g (Kuniko et al., 1989). In order to further analyze this, we fractionated the venom from *A. bilineatus* through gel filtration using a Sephadex G_75_ column (Supplementary Figure 2) and studied the neutralization of fraction A (mainly SVMPs) by sera 203 and 201. We observed that serum 203 (anti-*A. bilineatus*) was much better at neutralizing this fraction, which has an LD_50_ of 0.96 μg/g (ED_50_ = 15.6 mgV/mLS), than serum 201 (anti-*A. c. contortrix*) (ED_50_ < 0.9 mgV/mLS). Given that this fraction is mainly composed of Bilitoxin-I (data not shown), the neutralization difference provides strong evidence that the SVMPs in *A. c. contortrix* are immunochemically different from Bilitoxin-I, a finding that explains both the differences in neutralization potency of the tested sera against whole venoms and, at least in part, the differences in lethality.

It is important to note that a high ELISA titer does not necessarily reflect better neutralization. In fact, the sera with the highest titers were different from the ones with highest neutralization effective doses (Table 3).

The competitive ELISA experiments have also shown the presence of at least two groups with different immunochemical characteristics in *Agkistrodon* venoms, with the Mexican species in one and those from the U.S. in another. Additionally, the Mexican species appear to be more closely related to each other than *A. c. contortrix* and *A. piscivorus,* since these last two species show significantly lower competition percentage between them (Fig. 4).

Further observations can be made when neutralization potencies are studied in terms of the number of LD_50_s neutralized per mL of serum. When analyzed in that way, we can consider the variations in lethality of each venom. Interestingly, here, the venom of *A. russeolus* was better neutralized with all the analyzed sera. Previously, we mentioned that Bilitoxin-I is likely the main protein responsible for lethality of Mexican *Agkistrodon* venoms. Given that *A. russeolus* has approximately the same proportion of this toxin than *A. bilineatus* and *A. taylori* (16.8%, 15.3% and 17.5%, respectively), the higher lethal potency of the first could also be due to other toxins, which are less abundant in the latter. A good neutralization of these proteins by all the analyzed sera can be assumed, given their low effective dose against *A. russeolus* venom. However, a lot more studies should be performed to confirm this hypothesis.

The neutralizing potency of the equine monovalent sera generated in this work was equal to or higher than 1 mg of venom per milliliter of serum (1 mgV/mLS) for the heterologous venoms and higher than 2 mgV/mLS for the homologous venoms (Table 3). Judging from the experience of the authors, all sera have good neutralization potencies for antivenom production. Given that serum 203 (anti-*A. bilineatus*) was best at neutralizing the venoms from Mexican species and serum 202 (anti-*A. piscivorus*) was best at neutralizing all the analyzed venoms from the U.S., we consider that genus-wide neutralization can be achieved by the inclusion of venoms from *A. bilineatus* and *A. piscivorus* in equine immunization protocols.

## 5 Conclusion

Although proteomic analysis suggests that venoms are very similar in the composition of their protein families, our results demonstrate the presence of two groups in terms of immunochemistry and pharmacological activities. These differences have a significant influence on the cross neutralization of the produced sera. Therefore, the inclusion of one species from each group is important to generate a genus-wide neutralizing serum, efficient for treatment of envenomation by *Agkistrodon* species from both Mexico and the U.S.
