# Supplementary material for: Biochemical and immunochemical characterization of venoms from snakes of the genus Agkistrodon
Source: Toxicon X. 2019 Aug 2;4:100013. doi: 10.1016/j.toxcx.2019.100013 (PMC7285990; doi:10.1016/j.toxcx.2019.100013)
Supplement: Multimedia component 1 [file mmc1.pdf]

Supplementary information

Supplementary table 1. Equine hyperimmunization protocol.

	Immunization		Adjuvant		Venom		Blood sample
#	Date	Route	Type	Vol. (ml)	µg	µl	Vol. (ml)
1	18-Dec-13	*ID*	IFA	1.3	100	5	5
2	24-Dec-13	*SC*	IFA	0.7	100	5	5
3	08-Jan-14	*SC*	ALUM	0.4	200	10	5
4	22-Jan-14	*SC*	IFA	0.7	250	13	5
5	05-Feb-14	*SC*	ALUM	0.4	500	25	5
6	19-Feb-14	*SC*	-	0	1000	50	5
7	05-Mar-14	*SC*	IFA	0.7	2000	100	5
8	19-Mar-14	*SC*	ALUM	0.4	4000	200	250
9	02-Apr-14	*SC*	-	0	5000	250	250
10	16-Apr-14	*SC*	IFA	0.7	6000	300	250
11	30-Apr-14	*SC*	ALUM	0.4	6000	300	250
12	14-May-14	*SC*	-	0	6000	300	250

ID. Intradermal
SC. Subcutaneous
IFA. Incomplete freund adjuvant
ALUM. Alumina

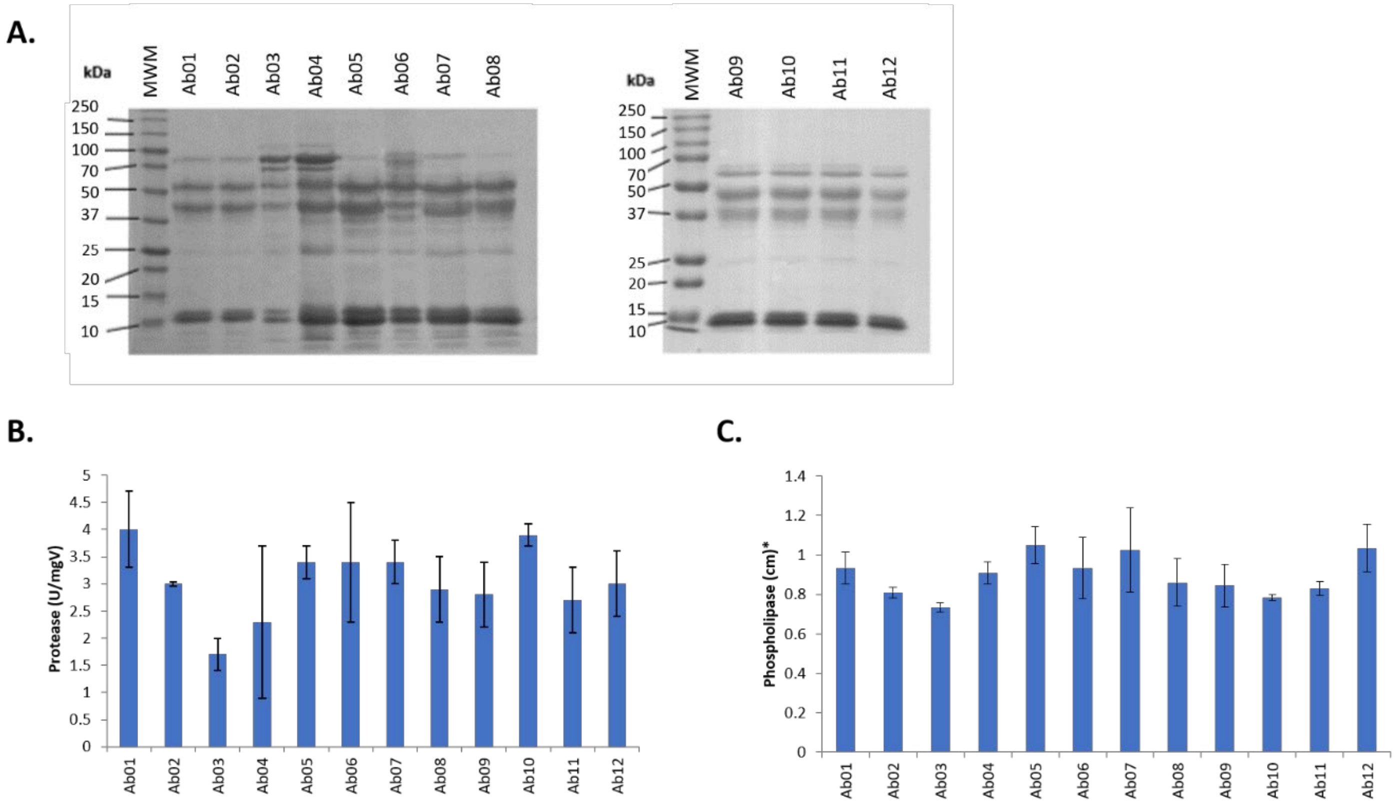

Supplementary figure 1. Venom variation among adult individuals of *Agkistrodon bilineatus* across its geographical distribution. A. 15% SDS-PAGE. *MWM.* Molecular weight markers. kDa. Kilodaltons. B. Protease activity on azocasein *U/mgV.* Units per milligram of venom. C. Phospholipase activity measured on agar plates and 10% egg-yolk solution as substrate. *cm.* Diameter of halo in centimeters. *Mexican state of origin of specimens: Ab01*. Colima. *Ab02*. Sinaloa. *Ab03, Ab11 and Ab12.* Morelos. *Ab04*. Nayarit. *Ab05*. Guerrero. *Ab06.* Oaxaca. Ab07-10. Chiapas.

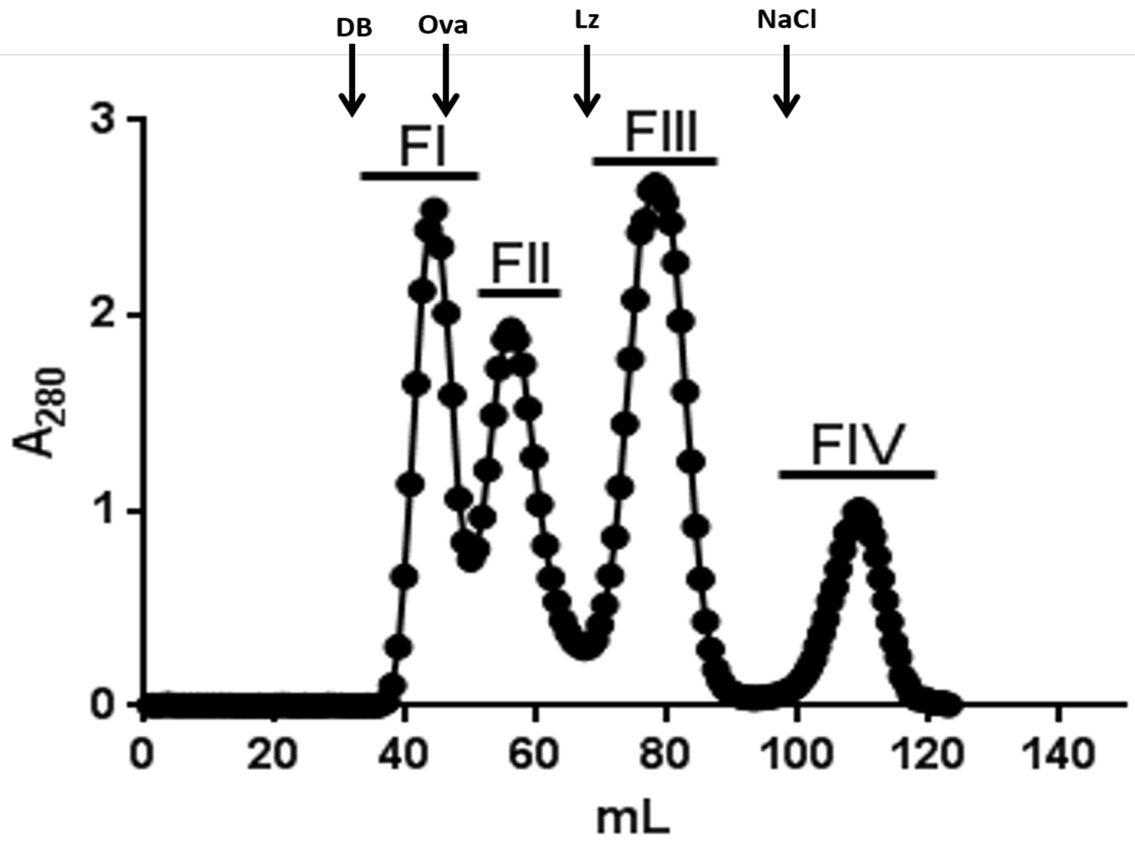

Supplementary figure 2. Sephadex G_{75} gel filtration chromatography of *Agkistrodon bilineatus* venom (80.5 AU_{280nm}). Column equilibrated with PBS, pH 7.2. Dimensions 197 x 90 mm. Total volume 125 mL. Flow speed: 14.2 mL/hr. Elution volume of molecular weight standards is shown with arrows. *DB.* Dextran blue. *Ova.* Ovoalbumin. Lz. Lizozyme. NaCl. Sodium chloride.
